# The 3D in vitro Adrenoid cell model recapitulates the complexity of the adrenal gland

**DOI:** 10.1038/s41598-024-58664-w

**Published:** 2024-04-05

**Authors:** Serena Martinelli, Giulia Cantini, Arianna Pia Propato, Daniele Bani, Daniele Guasti, Patrizia Nardini, Laura Calosi, Tommaso Mello, Nicole Bechmann, Giovanna Danza, Fabio Villanelli, Letizia Canu, Mario Maggi, Massimo Mannelli, Elena Rapizzi, Michaela Luconi

**Affiliations:** 1 Department of Experimental and Clinical Biomedical Sciences, University of Florence, 50139 Florence, Italy; 2 European Network for the Study of Adrenal Tumors (ENS@T) Center of Excellence, 50139 Florence, Italy; 3 Centro Di Ricerca E Innovazione Sulle Patologie Surrenaliche, AOU Careggi, 50139 Florence, Italy; 4 Department of Experimental and Clinical Medicine, Imaging Platform, University of Florence, 50139 Florence, Italy; 5 Institute of Clinical Chemistry and Laboratory Medicine, University Hospital Carl Gustav Carus, Medical Faculty Carl Gustav Carus, Technische Universität Dresden, Dresden, Germany; 6 Department of Experimental and Clinical Medicine, University of Florence, 50139 Florence, Italy

**Keywords:** Cancer models, Cell biology, Endocrinology, Cancer models, Fluorescence imaging

## Abstract

The crosstalk between the chromaffin and adrenocortical cells is essential for the endocrine activity of the adrenal glands. This interaction is also likely important for tumorigenesis and progression of adrenocortical cancer and pheochromocytoma. We developed a unique in vitro 3D model of the whole adrenal gland called Adrenoid consisting in adrenocortical carcinoma H295R and pheochromocytoma MTT cell lines. Adrenoids showed a round compact morphology with a growth rate significantly higher compared to MTT-spheroids. Confocal analysis of differential fluorescence staining of H295R and MTT cells demonstrated that H295R organized into small clusters inside Adrenoids dispersed in a core of MTT cells. Transmission electron microscopy confirmed the strict cell–cell interaction occurring between H295R and MTT cells in Adrenoids, which displayed ultrastructural features of more functional cells compared to the single cell type monolayer cultures. Adrenoid maintenance of the dual endocrine activity was demonstrated by the expression not only of cortical and chromaffin markers (steroidogenic factor 1, and chromogranin) but also by protein detection of the main enzymes involved in steroidogenesis (steroidogenic acute regulatory protein, and CYP11B1) and in catecholamine production (tyrosine hydroxylase and phenylethanolamine N-methyltransferase). Mass spectrometry detection of steroid hormones and liquid chromatography measurement of catecholamines confirmed Adrenoid functional activity. In conclusion, Adrenoids represent an innovative in vitro 3D-model that mimics the spatial and functional complexity of the adrenal gland, thus being a useful tool to investigate the crosstalk between the two endocrine components in the pathophysiology of this endocrine organ.

## Introduction

Three-dimensional (3D) cell culture has helped to transform the research in solid tumour modelling. These systems mimic the biochemical and cellular composition of tumours in their microenvironment more closely than 2D cultures. Specifically, 3D cultures can better reproduce the architectural organization of the tumour and the processes occurring in the developing and growing tumour mass, including hypoxia, necrosis, and cell–cell adhesion^1^.

The adrenal gland is an important regulator of homeostasis and stress response in the body. It is functionally and spatially organized into an outer cortex and an inner medulla. Embryologically, the catecholamine-producing medulla derives from the neural crest, while the steroidogenic cortex producing mineralocorticoids, glucocorticoids, and androgens, has a mesodermal origin. Even if the cortex and medulla have different embryological origins and a distinct endocrine secretion activity, they are physically and functionally interconnected showing several contact portions without separation by connective tissue or interstitial membranes^2^. Moreover, between the cortex and medulla, a portal circulation assures a strict exchange of locally produced-regulatory factors. It has been demonstrated that in the rat, some adrenal-medulla chromaffin cells are present showing adrenocortical mitochondria and smooth endoplasmic reticulum typical for steroid hormone producing cells^3^, thus providing the possibility for direct intercellular exchanges. Chromaffin cells have been observed within the cortex^2^ and, furthermore, islets of adrenocortical cells were found within the medulla^4,5^. During adrenal organogenesis, close interactions between the cortex and medulla are required for the correct morphogenesis and differentiation of the gland^6,7^, and this crosstalk also ensures the correct functionality of the adult adrenal gland. Adrenocortical glucocorticoids regulate chromaffin cell activity by stimulating tyrosine hydroxylase (TH) limiting enzyme expression rate in the catecholamine biosynthetic process^8^ as well as differentiation, by inducing the conversion of norepinephrine to epinephrine^9–12^. On the other hand, medullary neuroendocrine peptides have been described to stimulate cortex steroidogenesis in both in vitro and animal studies^2^. This two-way paracrine control is also supported by clinical observations, as glucocorticoid deficiency results in reduced plasma levels of catecholamines^13^, while in patients with adrenal medullary insufficiency due to the Shy-Drager syndrome, cortisol response to insulin-induced hypoglycaemia is abolished^14^. Given the physiological relevance of this complex interaction, it is also very likely an impact of the non-tumoral medullary and its cortical counterpart in adrenocortical carcinoma and in pheochromocytoma condition, respectively. To study the mechanisms underlying this intra-gland crosstalk as well as for drug testing, it is mandatory to develop a 3D multicellular spheroid structure consisting in both cortical and medullary cell types, which resembles the functionality of the organ and the complexity of interactions between the two cell types^7,15^.

In this work, we developed and characterized a unique 3D cell line model that recapitulates the complexity of the adrenal gland by mixing in vitro the mouse pheochromocytoma cell line, MTT, with the human adrenocortical carcinoma cell line, H295R. The resulting spheroid, which we called Adrenoid, mimicked the organization of the gland of origin and the distinct endocrine activity of medullary catecholamine and cortex corticosteroid secretion.

## Results

### Growth rate and gene expression of adrenal markers

**Figure 1:**
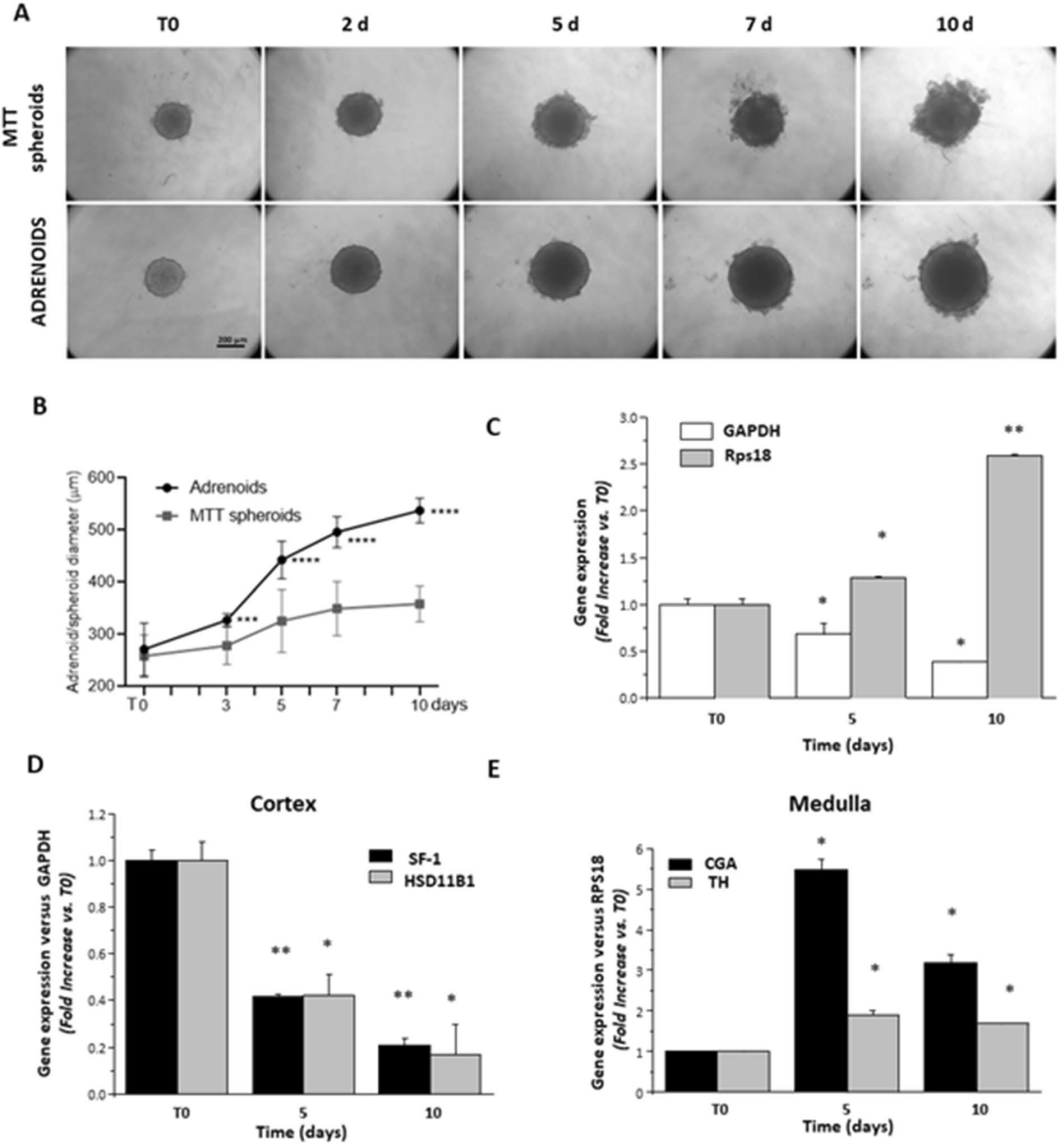
Adrenoid growth and cell composition. (**A**) Representative optical microscopy images of MTT spheroids and Adrenoids at different time points to monitor growth differences. (**B**) Diameters of MTT spheroids (squares) and of Adrenoids (circles) were measured at T0, 3, 5, 7 and 10 days and compared at each time point. Adrenoid diameters were significantly greater than those of the MTT spheroids starting from day 3, and by day 5 to day 10 the difference was even more significant. Graphs represent mean ± SD of diameters in three independent experiments performed in six replicates. Two-way ANOVA: ***p < 0.01 and ****p < 0.001. (**C**) Gene expression of the specific housekeeping markers (*Gapdh* and *Rsp18* for human H295R or mouse MTT cells, normalized on the other housekeeping gene, respectively) to assess the proportion of the two cell populations within the Adrenoid at 5 and 10 days. Bar graphs indicate gene expression mean ± SE fold increase on T0 in three independent experiments. An increased expression of *Rsp18* is associated with a decrease in *Gapdh* at the different time points, indicating an increased ratio between MTT and H295R cells over time; *p < 0.05 and **p < 0.001 vs T0, one-way ANOVA followed by Dunnett’s post hoc test. Gene expression of the cortex specific markers steroidogenic factor 1 (*Sf1*) and 11beta-hydroxysteroid dehydrogenase 1 (*Hsd11B1*) significantly decreased over time D), while gene expression of the medulla specific markers chromogranin a (*CgA*) and tyrosine hydroxylase (*Th*) significantly increased. Data are the mean ± SE fold increase of target gene expression normalized to the respective housekeeping gene (*Gapdh* and *Rps18* for human and mouse cell components, respectively) in three independent experiments; *p < 0.05 and **p < 0.001 vs T0, one-way ANOVA followed by Dunnett’s post hoc test.

To characterize Adrenoid growth, we compared the diameters of the mixed Adrenoids and of the MTT spheroids at different time points of culture starting from T0, corresponding to 48 h from 3D induction (Fig. 1A). The 2:1 ratio between MTT:H295R cells was chosen as the one that gave the best growth response and compactness of the 3D structure. Optimization of the Adrenoid culture conditions have been detailed in the Supplemental Data. Adrenoid growth rate was significantly higher than that of a MTT spheroid with the same initial number of cells starting from day 3 (*p* < 0.001); the difference in size increased at any of the following time points (day 5–7–10 *p* < 0.00001), as shown in Fig. 1B. Adrenoids maintained sharp margins up to day 10, indicating a strong interaction between the two cell populations to support Adrenoid compactness, limiting MTT cell high mobility evident in MTT spheroids (Fig. 1A). To assess the proportion between the medullary and cortical cells during the Adrenoid growth, we quantified the relative expression of two housekeeping genes specific for human (*Gapdh* for H295R) or murine (*Rps18* for MTT) cells at days 5 and 10. As shown in Fig. 1C, at day 5 and more evidently at day 10, there was a decrease in the human:mouse cell ratio, as shown by the significant reduction observed in *Gapdh* expression, normalized on the mouse housekeeping gene, and the corresponding increase in *Rps18* expression, normalized on the human housekeeping gene. Quantification of the Adrenoid expression of the adrenal cortex genes *Sf1* and *HSD11B1* on human cells (Fig. 1D), and of the adrenal medulla genes *CgA* (encoding chromogranin A) and *Th* on murine cells (Fig. 1E) showed a significant decrease in cortical and a significant increase in medullary markers at 5 and 10 days of Adrenoid culture. These findings suggest a modulation in the relative proportion of the cells, but also the maintenance of the expression of the functional markers characterizing the cortical and medullary lineage.

### Histology and ultrastructure morphology of Adrenoids

To investigate more deeply the Adrenoid organization and interaction occurring between cortical and medullary cells, we investigated 5-day-grown-Adrenoid by transmission electron microscopy (TEM) and compared the ultrastructure of MTT and H295R cells cultured together in the 3D structure with that observed in the single cell type monolayers.

**Figure 2:**
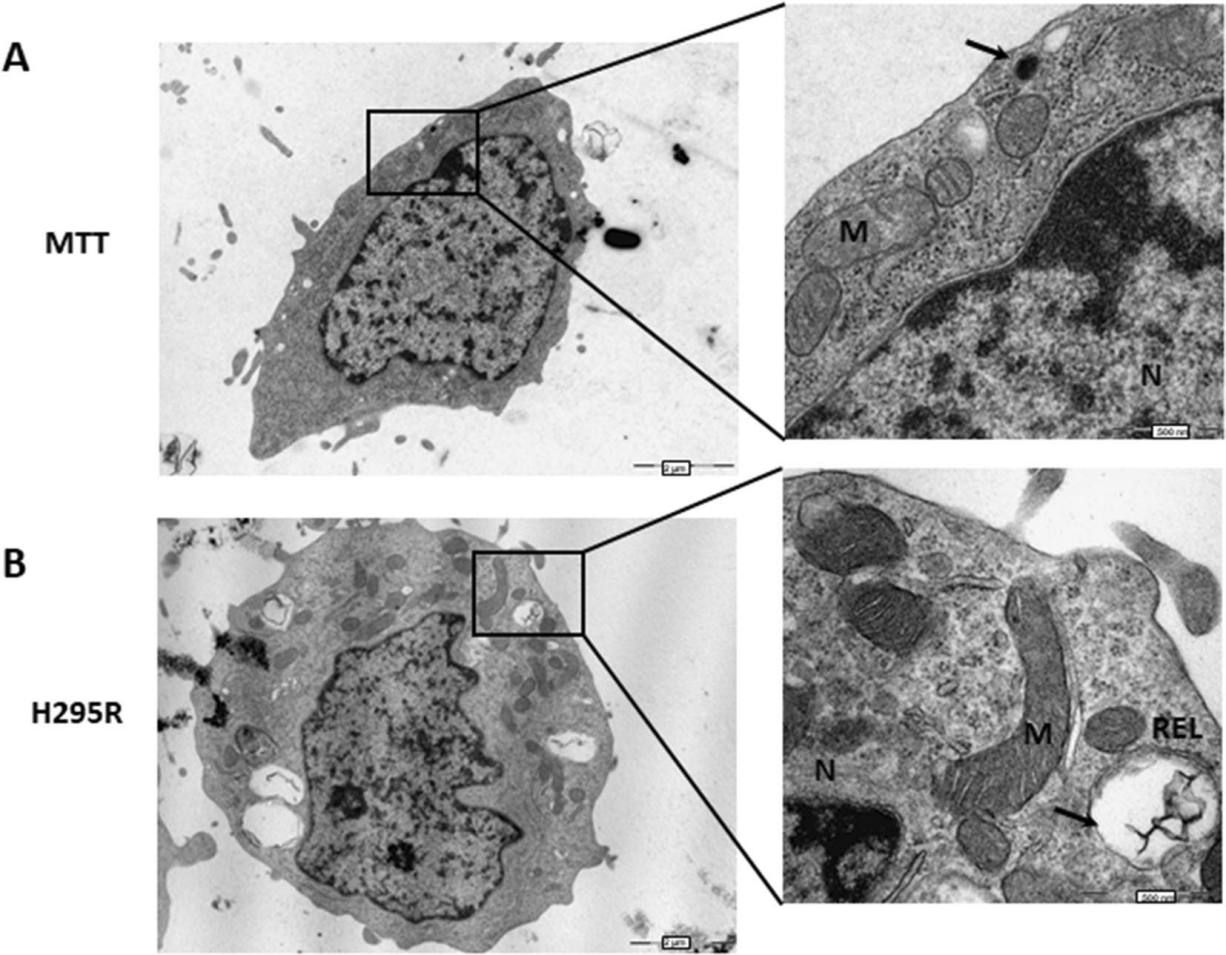
TEM analysis of cell ultrastructure of MTT and H295R grown as single cell type monolayers. (**A**) MTT cell grown in monolayer: the thin rim of cytosol poor of organelles and with few small chromaffin-like electron-dense granules indicates a poorly synthetic and active cell. Enlargement details the nucleus (N), mitochondria (M) and one putative catecholamine secretory granule (arrow). (**B**) H295R cell grown in monolayer: a magnification of cell showing the nucleus (N), mitochondria (M) and an electron-lucent vacuole resembling a liposome (arrow, REL), poor of organelles, indicating a rather steroidogenic immature stage. Scale bars are indicated at the right bottom.

**Figure 3:**
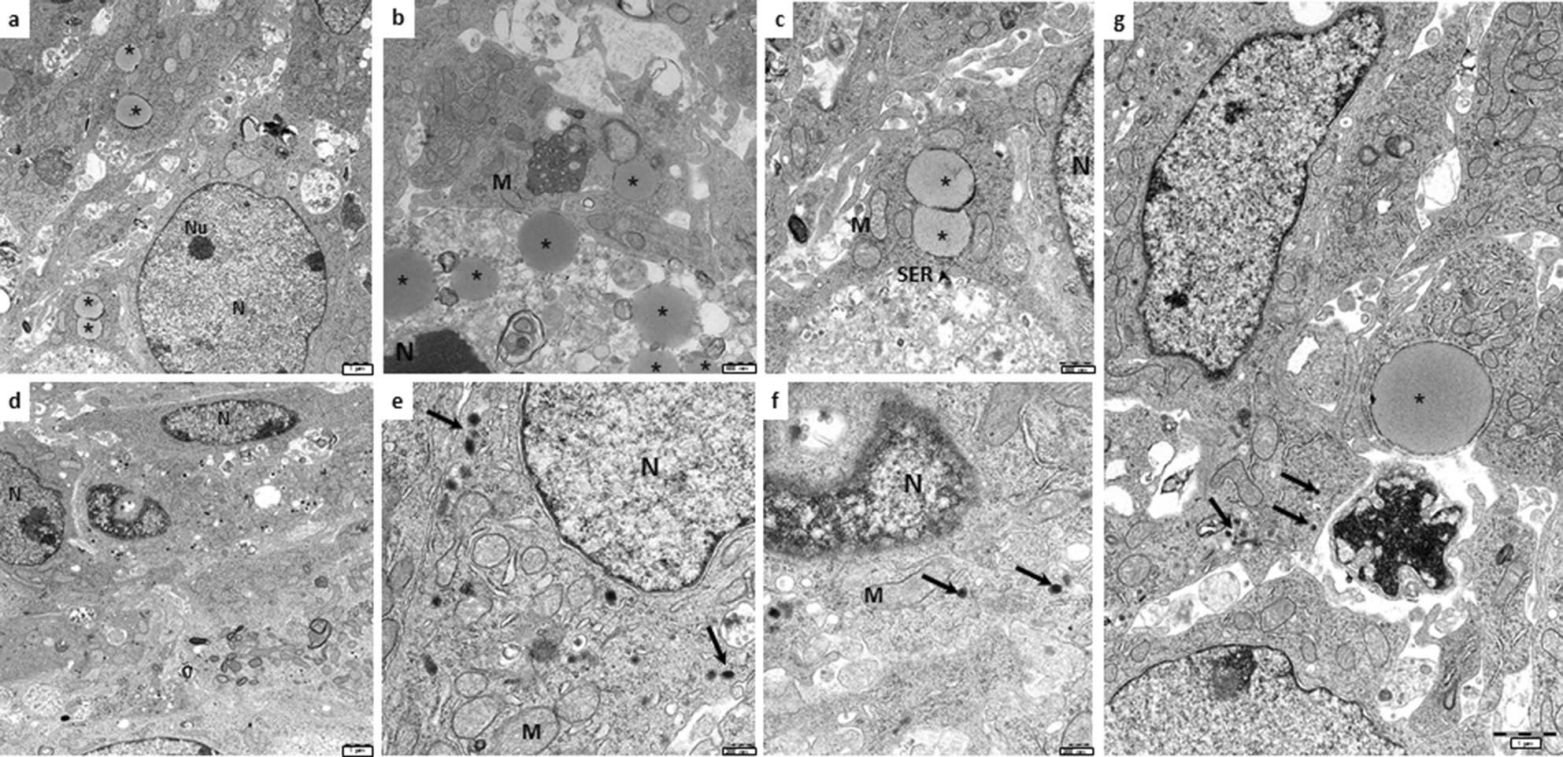
TEM analysis of Adrenoid ultrastructure. Representative TEM images of Adrenoids at day 5: (**a**–**c**) H295R cells resemble metabolically active cells, rich in mitochondria in proximity to typical lipid droplets (asterisk) and profiles of smooth endoplasmic reticulum (SER, arrowheads), suggesting the occurrence of cholesterol biosynthesis. (**d**–**f**) MTT cells within the Adrenoid showing several granules with electron-dense core and electron-lucent halo, closely resembling the typical catecholamine-containing secretion granules (arrows). (**g**) The picture shows an MTT cell (left), recognizable because of the dense-cored granules, in close contact with an H295R cell (right), characterized by the presence of a large lipid droplet (asterisk). Scale bars are indicated at the right bottom. Nucleus (N), nucleolus, (Nu), mitochondria (M), smooth endoplasmic reticulum (SER) are indicated.

**Figure 4:**
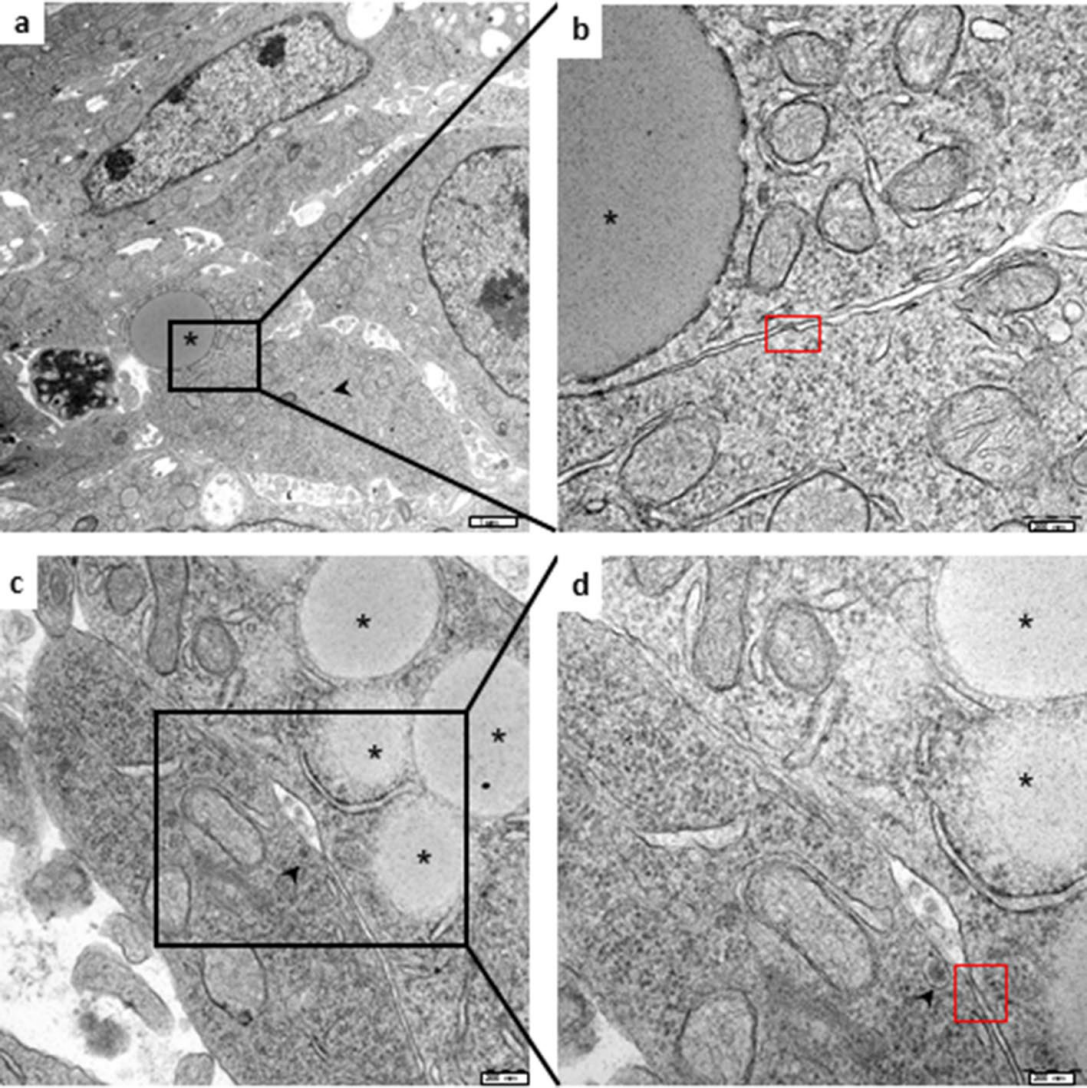
TEM analysis of MTT and H295R cell–cell junction in Adrenoids. Two examples of MTT and H295R cells recognizable by catecholamine-like granules (arrowheads) and a large lipid droplet (asterisks) respectively, in close contact within the Adrenoid. The enlargements show areas of plasma membrane juxtaposition and thickening (red boxes) suggesting the formation of specialized desmosome-like intercellular junctions between the two different cell types. Scale bars are indicated at the right bottom.

Figure 2A and B show representative TEM features of MTT and H295R cells grown as single cell type monolayers, respectively. MTT cells are characterized by the presence of electron-dense bodies resembling catecholamine-containing chromaffin secretion granules, like those described in pheochromocytomas^16^. On the other hand, H295R cells displayed the typical features of adrenal cortical cells with large, electron-lucent vacuoles resembling early liposomes surrounded by smooth endoplasmic reticulum and mitochondria, suggesting that steroid synthesis may occur. The cell features in the single cell type monolayers suggest that these cells display low synthetic activity, as indicated by the presence of few organelles, as well as the low number of chromaffin-like granules within the MTT and the immature liposomes in the H295R cells. When grown together into the Adrenoid, not only the morphology of the two cell populations was retained, but the cells displayed features of a higher synthetic activity than when they were grown alone in monotype (Fig. 3). H295R cells showed a greater number of mature liposomes and the presence of lipid droplets in respect to the same cells grown in monolayer culture (Fig. 3a–c). On the other side, MTT cells within the Adrenoid were richer of chromaffin-like secretory granules with typical dense cores and clear haloes (Fig. 3d–f) as compared with the same cells in homo monolayer culture. Intriguingly, in the Adrenoids, MTT and H295R cells were sometimes found in close contact by virtue of specific desmosome-like intercellular junctions (Figs. 3g and 4), which also account for a more functional adrenal-like phenotype.

### Cellular arrangement within Adrenoids over time

**Figure 5:**
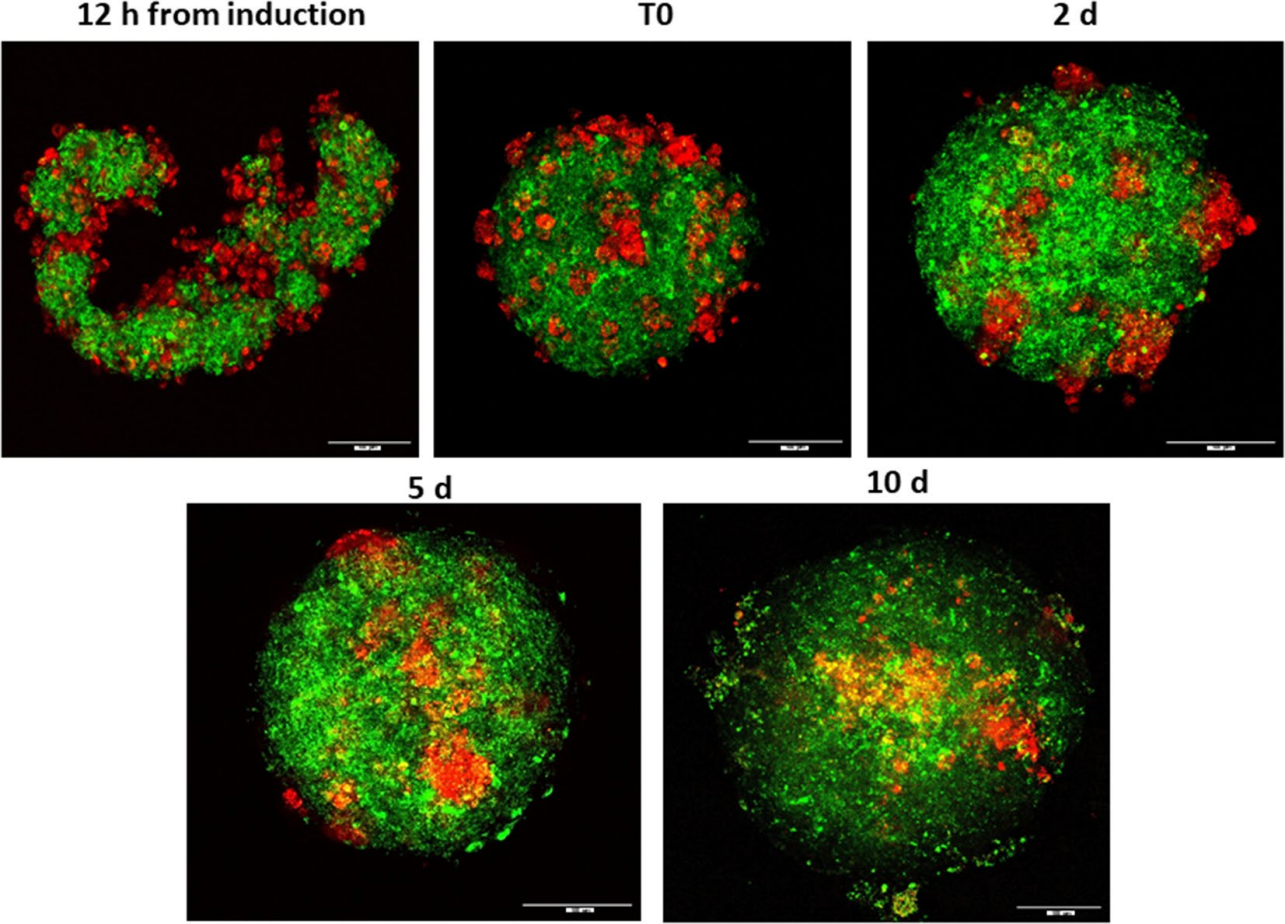
Fluorescence confocal microscopy analysis of spatial distribution of MTT and H295R cells inside the growing Adrenoid. Representative images of the Adrenoid at different time points of growth acquired by confocal microscopy. MTT and H295R cells were prestained with fluorescent viability staining (green and red, respectively) before generation of Adrenoids. Confocal images were taken through an HC PL 10x/0.30NA objective using sequential acquisitions of the green and red channels. Images are shown as the maximum intensity projection along the *z*-axis. The same Adrenoid was followed at 12 h from induction before transfer into chamber slide, and at T0 (48 h from induction) and at day 3, 5, 10 days after. The H295R cells tend to form clusters homogeneously diffused in an MTT core even before the formation of the compact 3D structure. From these images it is clear that Adrenoid growth is mainly due to MTT proliferation. Scale bars are indicated at the right bottom.

**Figure 6:**
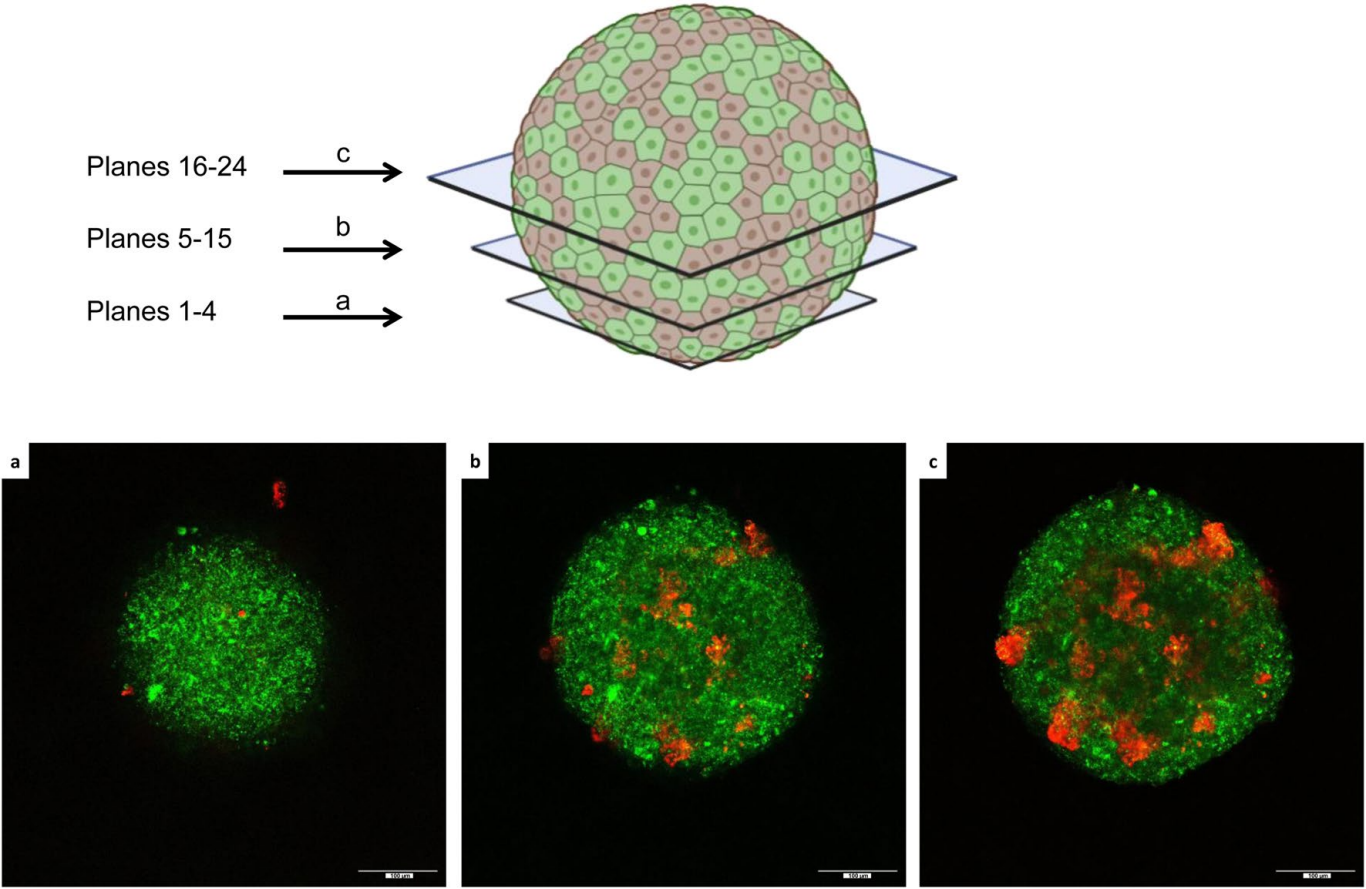
*Z*-axis distribution of H295R clusters and MTT cells inside the Adrenoid. (**A**) Different z-focal planes acquired by confocal microscopy in the lower hemisphere of the same Adrenoid at day 5 to better explore H295R cluster spatial distribution inside of the Adrenoid core. At the bottom of the Adrenoid most of the cells were MTT (prestained in green fluorescence) and very few, mostly single, H295R cells (prestained in red fluorescence) are appreciated (**a**, planes 1–4). Proceeding towards the central part of the Adrenoid (from plane 5–15) H295R cells tended to be organized in clusters, which are homogeneously distributed both in the centre and at the periphery of the Adrenoid (**b**). The homogenous distribution of H295R clusters was better appreciated in the central confocal sections of the Adrenoid (16–24 stack-planes) (**c**). Scale bars are indicated at the right bottom.

To follow the spontaneous cell organization during the Adrenoid growth, we marked MTT (green) and H295R (red) cells with vital fluorescent staining before Adrenoid induction and followed their spatial distribution over the time. Figure 5 shows the time course of Adrenoid growth: at 12 h from Adrenoid induction, the cells were randomly arranged in a structure that is not yet compacted in a spheroidal shape, while starting from T0 (48 h after induction), when the 3D structure has assumed a compact spheroidal morphology, H295R cells were scattered among MTT cells in the rounding Adrenoid. From day 3, H295R cell clusters appeared bigger as dispersed among MTT cells, making this aggregation even more evident at day 5. After additional 5 days of growth, at day 10, H295R clusters were still detectable embedded in the MTT population, despite the membrane labelling fading due to cell replication (Fig. 5). Figure 6 shows sequential stack images taken along *z*-axes of the Adrenoid at day 5 to better appreciate the spatial distribution of H295R clusters and MTT cells.

### Functional analysis of Adrenoids: enzymatic expression, catecholamine and corticosteroid production

**Figure 7:**
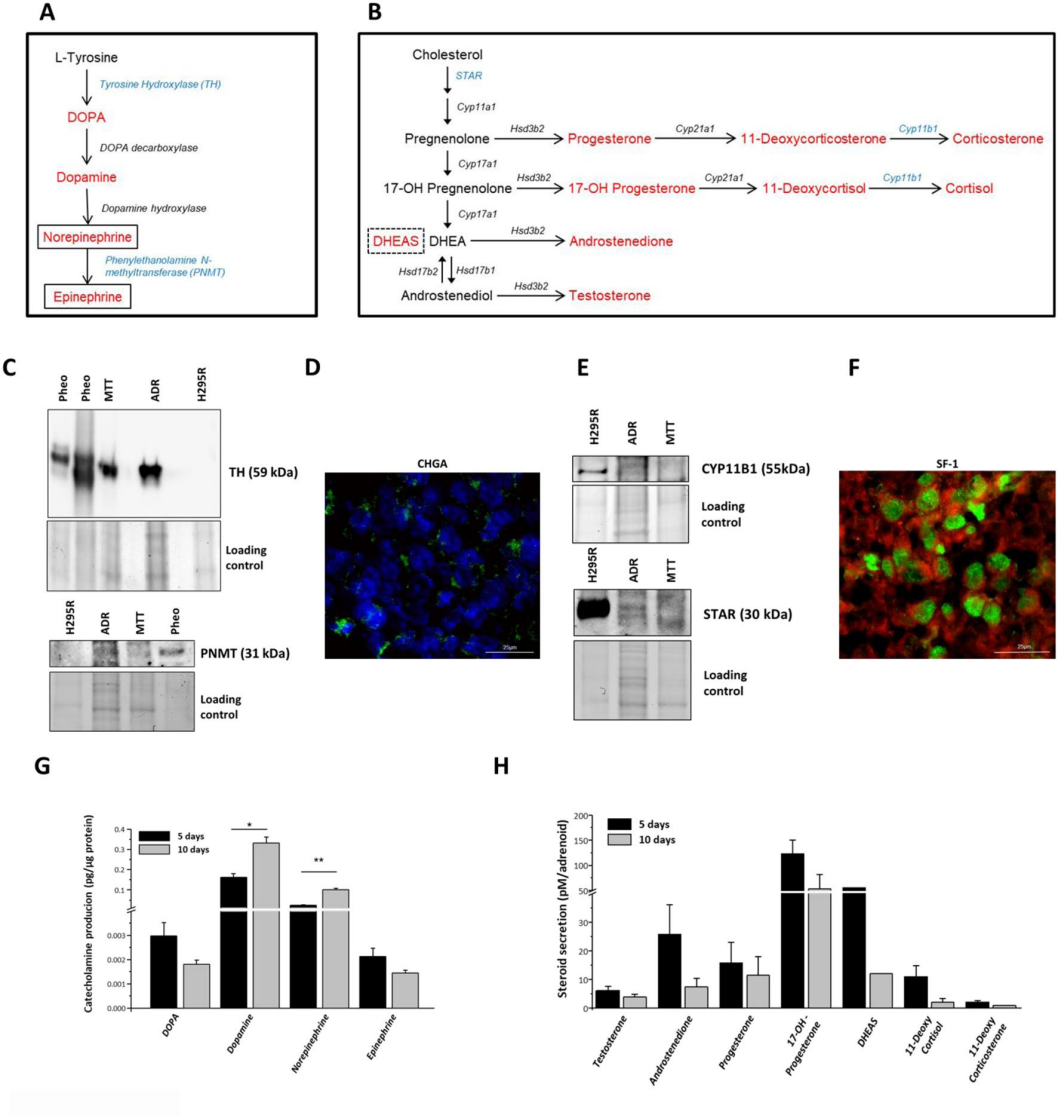
Dual endocrine activity of Adrenoids. Schematic representation of catecholamine (**A**) and steroidogenic (**B**) synthesis cascade: in blue are indicated the enzymes detected in Adrenoids by Western blot (WB, **C** and **E**); in red, the corresponding hormones. (**C**) Representative Western blot analysis of Tyrosine hydroxylase (TH) and phenylethanolamine N-methyltransferase (PNMT) protein expression in Adrenoids compared to positive (pheochromocytoma-Pheo-tissue lysates, MTT cells) and negative (H295R cells) controls. Molecular weight of the target proteins are shown in brackets. Stain-free gel total protein load/lane is shown as loading control. (**D**) Representative immunofluorescence staining for CHGA (green) at 40× magnification. Nuclear counterstaining is shown in blue. (**E**) Representative Western blot analysis of StAR and CYP11B1 proteins in Adrenoids compared to positive (H295R cells) and negative (MTT cells) controls. Stain free gel total protein load/lane is shown as loading control. For Western blots (panels **C** and **E**) to allow multiple detection of proteins on the same membranes, they were cut prior to hybridization with the different antibodies during the blotting procedure; moreover, acquired total images were further processed and cropped to evidence the target antigens. The corresponding fuller-length, original, unprocessed blots for each antibody and stain-free gels are shown in Fig. S8. (**F**) Representative image showing SF-1 nuclear expression detected by immunofluorescence staining (green) in H295R cell components of the Adrenoids, while MTT cells were traced by viability prestaining in red. Scale bar = 25 μm. (**G**) Catecholamine production at 5 and 10 days of the Adrenoid growth was measured intracellularly by LC-ECD. Bars are means ± SD of DOPA, dopamine, norepinephrine and epinephrine intracellular production normalized to the Adrenoid total protein content at the 2 time points in three independent experiments: *p < 0.05 and **p < 0.001, Student’s *t* test. (**H**) Steroid secretion was evaluated by mass spectrometry in conditioned media of Adrenoids at 5 and 10 days, normalized for the number of Adrenoids. Testosterone, androstenedione, progesterone, 17-OH-P, DHEAS, 11-deoxycortisol and 11-deoxycorticosterone were detectable at both day 5 and 10. Bars are means of steroid concentration ± SD/well in three independent experiments, except for DHEAS measurement performed in single.

To better study the functional activity of Adrenoids, some key enzymes of the catecholamine synthesis pathway and of the steroidogenic synthesis cascade have been evaluated (Fig. 7A and B, respectively). Western blot analysis of Adrenoids’ lysates compared to those obtained from MTT and H295R cell monolayers confirmed the presence of TH and phenylethanolamine N-methyltransferase (PNMT), as enzymes specific for the medulla (Fig. 7C), as well as the expression of CYP11B1 and StAR proteins specific for the cortical component (Fig. 7E). Immunofluorescence analysis of Adrenoids showed a positive signal for CHGA (Fig. 7D) at the cytoplasmatic level of chromaffin cells, and a strong nuclear positivity for SF-1 in cortical cells (Fig. 7F). Enzymatic activity of the Adrenoid was confirmed by measurement of catecholamine production (Fig. 7G) and steroid secretion (Fig. 7H). All hormones in the catecholamine synthesis pathway were detectable in Adrenoids, from DOPA to epinephrine (Fig. 7G). Comparing hormone production between Adrenoids and MTT spheroids cultured for 5 days in the same conditions revealed a more active synthesis in Adrenoids (fold increases = 1.5 for DOPA and dopamine, 1.2 for norepinephrine and 0.9 for epinephrine, where Adrenoid production was underestimated as normalized for total cell protein content), suggesting a positive effect of the cortical cells on stimulating catecholamine production.

Steroid secretion in Adrenoid conditioned media was measured by LC-mass spectrometry at the same time points of Adrenoid growth. All intermediate adrenocortical steroid hormones produced by glomerulosa (progesterone, 11-deoxycorticosterone), fasciculata (11-deoxycortisol, 17-OH-P), and reticularis (DHEAS, androstenedione and testosterone) adrenal zones, were detectable, except for cortisol (Fig. 7H). However, we derived the sensitivity for cortisol detection in our cell system model by measuring the steroid levels in conditioned media of H295R monolayers, resulting in a limit detection of 19.4 ± 6.3 ng/ml from 1 × 10^6^ cells. Of note, an increase in dopamine and norepinephrine was evident in Adrenoids between 5 and 10 days, but not in epinephrine, whose levels remained stable, while steroid secretion was reduced but still present, in accordance with the decrease ratio observed between H295R and MTT cells.

## Discussion

In this work, we have developed a unique 3D multicellular spheroid model, called Adrenoid, formed by cortical and medullary cells that resembles the organ functionality and the complex interactions occurring between the two endocrine components within the adrenal gland. Interestingly, the coexistence of cortico-steroidogenic and catecholamine secreting cells in the same gland during phylogenesis is exclusive of the mammalian adrenal glands^4^, suggesting an evolutionary advantage from the interaction between the two components. The communication between the chromaffin and adrenocortical cells that regulates the gland functions, including hormone synthesis and responses to stress^5,9^, may be relevant not only in physiology but also for the pathogenesis and progression of different diseases, including adrenal tumours^2^.

The 3D Adrenoids described in this work prove to be a valid and suitable model for future translational studies. In fact, it resembles the characteristics of the cells of origin in terms of functionality and growth, as well as in their 3D spatial interaction. The tumour origin of MTT and H295R cells not only allows in vitro cell growth, which is limited in primary normal cells, but also enables to study the contribution of the other adrenal component to the progression of each adrenal cancer type, pheochromocytoma and adrenocortical carcinoma. This is not possible when cells are grown in single monolayers. By mixing the two cell lines, we were able to obtain a 3D structure where the neuroendocrine cells in particular take a growth advantage compared to spheroids composed by MTT only, suggesting the positive effect exerted by cortical cells on medullary cell proliferation. Despite the several set-up passages we tested (detailed in the Supplemental Data), the culture conditions of the Adrenoid were not yet optimized for H295R growth, as cortical cell ratio in the 3D structure tended to decrease over time, despite the remaining cell maintenance of steroidogenic activity. Notably, TEM ultrastructure analysis of Adrenoids showed that inside the 3D structures close cell–cell interactions occurred between MTT and H295R cells, associated with a more active and synthetic phenotype compared with their single cell culture monolayer counterparts. In fact, in the Adrenoids, their cytoplasm was richer in mitochondria and organelles, in particular in liposomes and mature lipid droplets associated with expanded SER and numerous mitochondria in H295R cells, and in dense secretory granules in MTT cells, all being specific features of the two cell types, suggesting a more metabolically active status of the cells when they are grown together in 3D. Mass spectrometry analysis of steroid secretion and LC-ECD measurement of catecholamine production from Adrenoids confirmed their morphological and ultrastructure features, being the metabolomic approach a pivotal tool of analysis of functionality in adrenal models and pathologies^17^. Indeed, Adrenoids displayed production of all types of catecholamines from DOPA to epinephrine, with a relevant ratio between norepinephrine and epinephrine, typical of pheochromocytoma tumours and pheo cell lines, compared to normal adrenals^16,18,19^. In fact, very few pheochromocytomas secrete epinephrine compared with those that secrete norepinephrine^20^. The Adrenoids also expressed high levels of TH and low levels of PNMT, key enzymes of catecholamines synthesis. These results are in line with Isobe et al. who found lower concentrations of *PNMT* mRNA in pheochromocytoma compared with normal adrenal medulla, explained by lower concentrations of cortisol reaching tumor tissues^21,22^.

Interestingly, catecholamine production from MTT cells was higher when they form Adrenoids with H295R compared to MTT spheroids, suggesting that MTT cells are more active when they interact with H295R cells than when alone in spheroids, confirming a positive crosstalk between medullary and cortical cells^8,23^.We also demonstrated that Adrenoids retained corticosteroid secretion activity, although cortisol was not detectable, probably because of the low number of cortical cells present within the Adrenoid. All other steroids were detected, even considering the low number of H295R cells within the 3D structure. Intriguingly, steroids dosage showed high levels of intermediates, such as 17-OH-Progesterone and Progesterone, and low levels of glucocorticoids in the later stages, such as 11-Deoxycortisol and 11-Deoxycorticosterone, suggesting that steroidogenesis was present but was not stimulated toward the final stages of glucocorticoid production. We observed a decrease in steroids levels between 10 and 5 days, probably due to the reduction in the ratio of H295R to MTT during the Adrenoid culture. Adrenoid growth may be, however, overestimated due to increased ratio between MTT and H295R composition occurring during Adrenoid growth. Moreover, the initial 2:1 ratio between MTT and H295R cells could influence the subsequent decrease observed in H295R cells in the days that follow 3D induction. In physiological conditions, in fact, the relationship between the two cellular components should be opposite. To set up our model, we tried combinations of different conditions including centrifugation methods, ratio of the cells, number of cells and culture medium; at last, we chose this proportion as it gave the best results in terms of speed of spheroid compacting (see details in Supplemental Data). Reverse proportions will be explored in the future for optimizing the growth conditions for the cortical component, as well as implementation of the culture media able to support MTT growth. Since H295R cells are not representative of a single cortical cell type and their steroid hormone secretion profile has been described to vary according to laboratory strain, passage, and growing conditions^24–26^, differences in steroid production levels may be likely. Interestingly, in the Adrenoids all the three steroidogenic production is present as demonstrated by detection of intermediate steroids typical of glomerulosa, fasciculata and reticularis. In particular, the mix condition occurring in the Adrenoids where H295R are grown with mouse MTT, and the culture medium is optimized for MTT spheroids, not including specific factors used for H295R culture^18,27^ may affect the final step of cortisol synthesis. The use of mouse MTT cells with human H295R cells to form the Adrenoids may represent a limitation of the model, as putative off-target interspecies effects may occur. However, the different species origin of the cortical and medullary cells allowed us to better follow the two cellular components experimentally (Fig. 1C and D). Moreover, MTT and MPC mouse pheochromocytoma cells are currently the only catecholamine secreting tumour cell line available and well characterized^16^. Secreting cell lines derived from human pheochromocytomas have not yet been developed, as hPheo1 human cell line lacks hormone production and the genes associated with catecholamines synthesis are downregulated^28,29^. Moreover, considering that corticosterone is the most represented among the corticosteroids of rodents, in the Adrenoid there could be different interaction mechanisms compared to the in-vivo situation, which might result in very low production of cortisol by H295R cells^26^. In addition, the low number of H295R cells present in the Adrenoids probably does not allow to measure cortisol, which was detectable only when a 10^3^-fold higher number of H295R were grown in monolayer conditions.

To date, several attempts have been performed to induce homo-cell spheroids from H295R cells with different methods with variable efficacy^30–35^, showing different cell viability and growth rate. In our hands, homotypic spheroids obtained by H295R cells were not able to either compact properly and maintain steroidogenic activity, as shown in the Supplemental Data. Indeed, none of the currently available monotypic H295R-3D structures have been characterized for their complete steroidogenic production panel, except for aldosterone^32^ and cortisol^35^ secretion. In addition, the only functional 3D mixed structure so far developed in vitro from human cell populations is represented by the adrenal organoid obtained from mixed primary cell populations obtained from foetal adrenals between week 9th and 12th of gestation, where the coexistence of both neuroendocrine and steroidogenic cells in close contact assured not only the functional activity, but also the spatial distribution, thus resembling the organ of origin. The Adrenoid obtained in the present study from MTT and H295R cell co-culture is the first 3D adult adrenocortical cell model where steroidogenic activity is maintained, clearly demonstrating the requirement of the presence of the medullary adrenal component.

When we examined the cellular organisation within the Adrenoid, we observed that H295R cells tended to aggregate and form clusters within the MTT cell mass, disposing not only on the surface but also in the internal part of the 3D structure. This could be explained in part by the fact that in vivo the proportion of cortical cells is much greater than the amount of medullary cells, contrary to the in vitro Adrenoid ratio. Furthermore, the adrenal capsule has been demonstrated to recruit cells to the steroidogenic lineage and regulate spatial and functional organization among the three concentric cortical zones. In our model, the lack of a capsule could lead to a cell recruitment defect and, consequently, to an altered organization of the adrenal cortex component^36,37^. Moreover, the lack of blood vessels inside the 3D mass could also affect the H295R distribution: the adrenal gland is one of the most vascularised organ and its vascular network plays an important role not only during embryogenesis, but also throughout life to provide precursors for the steroidogenesis and to allow hormones secretion in the blood flow^38^. The specific branching of the adrenal vasculature suggests strong interactions between endothelial and adrenal cells^39^, allowing for their coordinated functions. The missing interaction between the endocrine (present) and endothelial (not present) cells within the Adrenoid could finally affect the spatial organisation of the cortical cells. Implementation of the Adrenoid structure by adding capsular and endothelial cells will clarify these aspects and overcome the limit of our model.

In conclusion, the Adrenoid we developed represents an innovative in vitro complex 3D model that mimics the functional complexity of the adrenal gland. Once optimized, it will be useful to extend the repertoire of preclinical models of endocrine tumours for investigating the cellular mechanisms of adrenal cancer and testing of novel drugs.

## Methods and materials

### Cell cultures

The mouse pheochromocytoma tumour-derived (MTT) cell line^16^, kindly provided by Arthur Tischler, was grown in DMEM with 10% FBS (Fetal Bovine Serum, Sigma-Aldrich), 2 mM L-glutamine, 100 U/ml penicillin-100 μg/ml streptomycin, supplemented with 5% horse serum (HS). Human ACC cell line NCI-H295R (H295R) was obtained from the American Type Culture Collection (Manassas, VA, USA) and was cultured in DMEM/F12 medium (Sigma-Aldrich, Milan, Italy) with 10% FBS, 2 mM L-glutamine, 100 U/ml penicillin and 100 μg/ml streptomycin, which was further enriched with a mixture of insulin/transferrin/selenium (Sigma-Aldrich). In both cases cells were grown at 37 °C in a 5% CO_2_ humidified atmosphere.

### Adrenoid and spheroid induction and growth

Tumour cells were grown as adherent monolayer cultures until confluence and dissociated into single cells. To generate Adrenoids from these primary tumour-derived secreting cells, several approaches have been used and reported in details in the Supplemental Data. As the best adopted protocol, a total of 2 × 10^3^ cells in a 2:1 ratio of MTT and H295R cells, respectively, were added into each well of a non-adhesive round bottomed 96-well plate (Nunclon Sphera, Thermo Fisher); the 2:1 ratio was chosen as the best for Adrenoid compacting. To generate spheroids of single cell type, 2 × 10^3^ MTT cells were finally adopted. The round bottomed plates were then centrifuged at 1000×*g* at room temperature (RT) for 10 min to initiate cell–cell interaction and incubated at 37 °C, 5% CO_2_. This procedure generated Adrenoids and spheroids with homogeneous size and geometry. After 48 h from induction, Adrenoids and spheroids showed a round morphology with clear margins and were considered as T0^40^ for all further experiments. Adrenoids and spheroids were maintained in DMEM supplemented with 10% FBS which was chosen after different experiments performed to optimize the medium for the mixed culture (Supplemental Data). This is the medium usually used for MTT-spheroid cultures^15,40^, and for monolayer experiments with H295R^41^, except for some growth supplementations^27^. To assess Adrenoid and spheroid growth, culture medium was replaced with fresh medium at T0, and images were acquired at different time points (T0, 3, 5, 7 and 10 days), and diameters were measured with ImageJ software^42^.

### RNA isolation and quantitative real-time RT-PCR (qRT-PCR)

RNA isolation and quantitative real-time RT-PCR (qRT-PCR) were performed as detailed elsewhere^43^ using specific Taqman Gene Expression Assay on Demand (Life technologies) for the following genes: *Gapdh* FAM-MGB (glyceraldehyde-3-phosphate dehydrogenase, #4352934), human *HSD11B1* (hydroxysteroid 11-beta dehydrogenase 1, #Hs01547870_m1), human *NR5A1* (SF1, steroidogenic factor 1, #Hs00610436_m1), mouse *Th* (tyrosine hydroxylase, #Mm00447557_m1), mouse *Cga* (chromogranin A, #Mm00514341_m1), mouse *Rps18* (ribosomal protein S18, #Mm02601777_g1). The amount of target, normalized to an endogenous reference gene, *Gapdh* for human genes or mouse *Rps18* for mouse genes, and relative to a calibrator (Stratagene, La Jolla, CA, USA) was expressed by 2^−ΔΔCt^ calculation.

### Trasmission electron microscopy

Monolayer cultured cells and Adrenoids at day 5 were washed with PBS and were directly fixed in Karnovsky fixative overnight at 4 °C and post fixed in 1% osmium tetroxide in 0.1 M phosphate buffer (pH 7.4) for 1 h RT^7^. The samples were dehydrated in graded acetone, passed through propylene oxide, and embedded in epoxy resin. Ultrathin sections were stained with gadolinium acetate and alkaline bismuth subnitrate and examined under a JEM 1010 electron microscope (Jeol, Tokyo, Japan) at 80 kV. Photomicrographs were taken with a MegaView III (Soft Imaging System, Muenster, Germany) digital camera connected with a dedicated software (AnalySIS, Soft Imaging Software, Muenster, Germany).

### Confocal microscopy of 3D Adrenoids

To discriminate the two cell populations (MTT or H295R) within the Adrenoids, two fluorescent viability dyes with long aliphatic tails were incorporated into lipid regions of the cell membrane. PKH26 Red Fluorescent Cell Linker Kit and PKH67 Green Fluorescent Cell Linker Kit were purchased from Sigma-Aldrich (St. Louis, MO; Cat. no. PKH26GL and PKH67GL, respectively). Cells were labelled according to the manufacturer’s instructions. Briefly, a total of 1 × 10^6^ cells were washed and suspended with serum-free DMEM. After centrifugation at 400×*g* for 5 min, supernatant was discarded. H295R cells and MTT cells were stained with solution C containing PKH26 or PKH67 staining reagents, respectively. The mixture was incubated at 25 °C for 2–5 min and was gently mixed by rocking the tube forward and backward during the incubation period. The staining action was blocked by adding the same volume of FBS for 1-min incubation. Then, cells were centrifuged at 400×*g* for 10 min at 25 °C. The supernatant was removed, and the cells were washed three times in 10 mL of complete culture medium. Then, cells were adjusted to an appropriate density and used for Adrenoid induction. After induction, images of Adrenoid were acquired at 12 h, and at T0 (48 h from induction) and at the following day 3, 5, 10 by using a Leica SP8 confocal microscope (Leica Microsystems). Images were collected through a HC PL 10x/0.30NA objective using sequential acquisitions of the green (excitation 571 nm, emission 493–523 nm) and red (excitation 551 nm, emission 557–586 nm) channels and system optimized voxel size (XY = 0.331 µm, Z = 4.28 µm). Images were prepared for publication using the Fiji software^42^ and are shown as maximum intensity projection along the *z*-axis.

### Sodium dodecyl sulfate–polyacrylamide gel electrophoresis (SDS‑PAGE) and Western blot analysis of proteins

Protein lysates were obtained as previously detailed^44^. Proteins derived from tissue/cell/Adrenoids were extracted in RIPA buffer (20 mM Tris, pH 7.4, 150 mM NaCl, 0.5% Triton-100, 1 mM Na_3_VO_4_, 1 mM PMSF) after repeated freezing and thawing cycles, at least three times, followed by a sonication step for 30 s at maximum power (Ultrasonic Cleaner, VWR International, Milan, Italy). After protein content measurement, protein lysates were separated by reducing SDS-PAGE stain-free precast gels (Bio-Rad) and transferred to polyvinylidenedifluoride (PVDF) membranes (Trans-BlotTurbo, Bio-Rad). Fifty micrograms were loaded for Adrenoid lysates, while twenty-five micrograms for positive and negative controls consisting of monolayer MTT and H295R cells and pheochromocytoma tissue obtained from the Repository of biological samples of the European Network for the Study of Adrenal Tumours (Ethical Committee approval-Prot. 2011/0020149—Rif CEAVC Em. 2019-201 26/11/2019).

The blots were cut prior to hybridisation with specific antibodies according to the protein molecular weight. Cut-out membranes were probed with the following primary antibodies, after a 30-min blocking phase in 3% non-fat dried milk (ITW Reagents, S.R.L., Monza, Italy): Anti-StAR (1:1000, sc-25806; Santa Cruz Biotechnology, Ca; USA), Anti-CYP11B1 (1:1000, #A7664, Abclonal, Woburn, MA, USA); Anti-CYP11B1 (1:1000, sc-377401, Santa Cruz Biotechnology, Ca; USA), anti-PNMT (1:1000, #A20862, Abclonal, Woburn, MA, USA), anti-TH (1:1000, #A5079, Abclonal, Woburn, MA, USA).

Each membrane was incubated overnight at 4 °C with primary antibodies followed by peroxidase-conjugated secondary IgGs (1:3000). Image acquisition and analysis were performed with Image Lab software version 6.0 on a Chemi-Doc TM Touch instrument (Bio-Rad), using fluorescence emission of protein bands separated on stain-free gels for the total lane normalization^44,45^.

### Immunofluorescence staining of Adrenoids

To perform immunofluorescence staining of cortical and medullary components in Adrenoids, we used primary antibodies against SF-1 and CHGA on Adrenoids obtained with MTT cells vitally stained by PKH26 Red Fluorescent Cell Linker Kit (as described above) and H295R cells. After 5 days of culture, Adrenoids were included in O.C.T. Compound Embedding medium for cryostat (Bio-Optica, Milan, Italy), quickly frozen at − 80 °C and sectioned by cryotome. Cryosections of 7 µm were fixed as previously described: briefly, Adrenoids were fixed with 4% paraformaldehyde, for 10 min at room temperature (RT)^46^. The sections were then washed in PBS, then permeabilized in 0.2% Triton-X100 in PBS for 20 min RT, then blocked with 2% bovine serum albumin (BSA, Applichem, Darmastad, Germany) in PBS for 1 h at RT to minimize non-specific binding. The primary antibodies (anti-SF-1, 1:100, #07–618, Upstate, Sigma-Aldrich and anti-CHGA, 1:50, ThermoFisher) were diluted in BSA 2% PBS and 0.2% Triton-X100 and incubated overnight at 4 °C. The next day, the sections were incubated for 2 h at RT in the dark with appropriate fluorochrome-conjugated secondary antibodies diluted in BSA 2% PBS (Alexa Fluor anti-mouse & anti-rabbit 488, Jackson ImmunoResearch Labs, West Grove, PA, USA). Nuclear counterstaining was obtained through incubation with DAPI for 1 h at RT. Subsequently, the specimens were rinsed three times with PBS and then mounted with Fluoromount aqueous medium (Sigma-Aldrich). Images were acquired with a Leica DM4000 epifluorescence microscope (Leica Microsystems GmbH).

### Catecholamines measurements

Adrenoids were cultured for 5 or 10 days from T0. Afterwards, Adrenoids (*n* = 36) were collected, washed with PBS and stored at − 80 °C. Lysis was performed as previously described^47^ using 60 µL of cell disruption buffer. After centrifugation, 40 µL of the collected supernatant was dissolved with 60 µL perchloric acid and analysed by liquid chromatography with electrochemical detection (LC-ECD)^47,48^. The protein amount was quantified by Bradford assay^47^. MTT spheroids (*n* = 36) cultured in the same conditions for 5 days as a positive control for catecholamine secretion.

### Steroids evaluation by mass spectrometry

Ninety-six Adrenoids were cultured for 5 and 10 days from T0 in media without phenol red. Twenty-four hours before the established time points, all Adrenoids were transferred to a single well, a washout step was performed, and 200 µL of serum-free medium without phenol red was added. The collected media were stored at − 20 °C until steroid analysis was performed.

Steroids were quantified by liquid chromatography-tandem mass spectrometry (LC–MS/MS), using a SCIEX QTRAP 6500 instrument (Sciex, Framingham, MA), with ESI source, interfaced with a high-pressure liquid chromatography, HPLC 1260 (Agilent Technologies, Santa Clara, CA) equipped with an isocratic pump for sample loading, a binary pump for chromatographic separation and a thermostated autosampler. The column oven temperature was maintained at 40 °C. Briefly, 100 μL of sample were added to 200 μL of internal standard solution in CH3CN with formic acid 0.1% (v/v), precipitated proteins were separated by centrifugation for 10′ at 14,000 rpm at room temperature, the supernatant was diluted adding 700 μL of deionized H2O and 30 μL of this solution were injected. Internal standards were isotopically labelled analytes (13C or deuterium labeled) purchased from Sigma-Aldrich (St. Louis, MO). Chromatographic separation was performed using a SPE C18 5 μm 20 × 2 mm pre-concentrating washing column and a Kinetex C18 3 μm 100 × 3 mm analytical column (Phenomenex, Torrance, CA) Elution solvents were water with 0.2 mM of Ammonium Fluoride (solvent A) and methanol (solvent B) and the elution gradient was from 43 to 98% of solvent B in 10 min. The mass spectrometer operated in Multiple Reaction Monitoring (MRM) mode with ESI source, in positive ion mode for analytes A, T, P, 17-OH-P, DHEA, E, and F, 11-Deoxycortisol, 11-Deoxycorticosterone and negative for DHEAS. Acquired transitions were the following (two product ions for each analyte): Androstenedione (A) 287-97, 287-09; Testosterone (T) 289-97, 289-109; Progesterone (P) 315-97, 315-109; 17-Hydroxyprogesterone (17-OH-P) 331-97, 331-109; Dehydroepiandrosterone Sulfate (DHEAS) 367-97; Cortisol (F) 363-121, 363-97; Corticosterone (B) 347-121, 347-91; 11-DeoxyCortisol 347-97, 347-109; 11-Deoxycorticosterone 331-97, 331-109. A multilevel serum calibrator set was used (Chromsystems steroid panel 1 + 2) for quantification that was performed using MultiQuant software (Sciex).

### Statistical analysis

Data analyses were performed by GraphPad Prism Version 8.3.0 for Windows (GraphPad Software). Results are expressed as mean ± SD (standard deviation) or ± SE (standard error) as detailed in each figure legend. Comparisons of means between two groups of data were performed by Student’s t-test, multiple comparisons by One-way ANOVA followed by Dunnett’s post hoc test or Two-way ANOVA, with *P* < 0.05 taken to be statistically significant.

### Supplementary Information

Supplementary Information.

## Data Availability

All data generated or analysed during this study are included in this published article [and its supplementary information files]. The authors declare their compliance with the digital image and integrity policies (https://www.nature.com/srep/journal-policies/editorial-policies#digital-image).
