# Supplementary material for: The 3D in vitro Adrenoid cell model recapitulates the complexity of the adrenal gland
Source: Sci Rep. 2024 Apr 5;14:8044. doi: 10.1038/s41598-024-58664-w (PMC10997590; doi:10.1038/s41598-024-58664-w)

Fig. S1: Different optimization steps for the Adrenoid formation

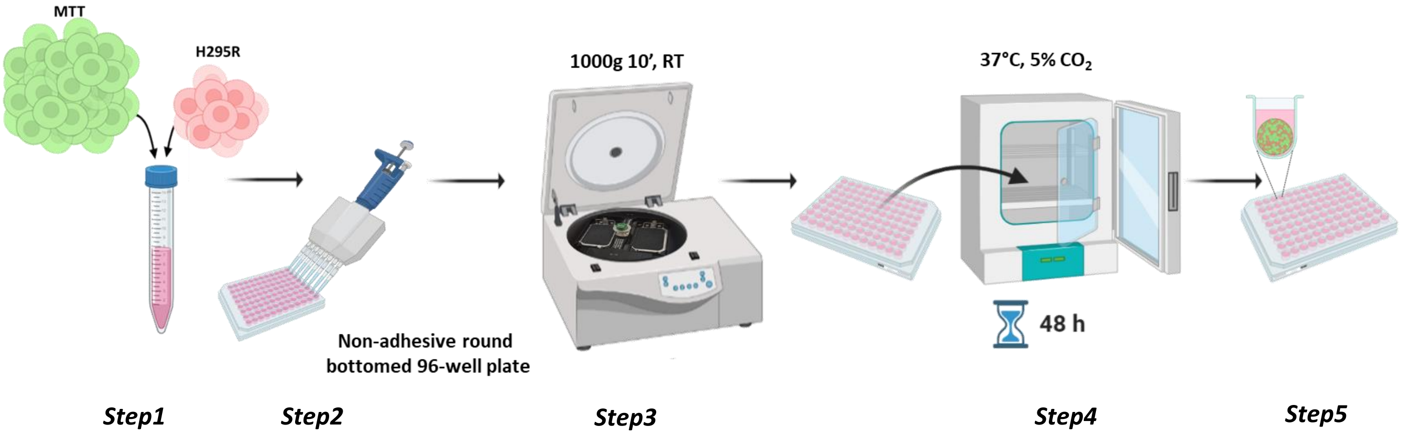

	Set-up conditions	Figure	BEST SET - UP
MTT:H295R Ratio *Step1*	1:1<br>2:1<br>3:1<br>1:2	*Fig. S2, Fig. S5*	**2:1**
Plating total Cell Number *Step2*	2000<br>5000<br>10000	*Fig. S2, Fig. S6*	**2000**
Aggregation method *Step3*	1) Settling	*Fig. S3*	**Centrifugation**
	2) Orbital shaker (2 hours)	*Fig. S4*	
	3) Centrifugation	*Fig. S2, Fig. S5*	
Speed/Timing of centrifugation *Step3*	220g - 10 min<br>1000g - 5 min<br>1000g - 10 min	*Fig. S2, Fig. S5, Fig. S6*	**1000g – 10 min**
Timing of 3D formation (hours) *Step4*	24<br>48<br>72<br>96	*Fig. S6*	**48**
Culture Medium *Step5*	▪ DMEM + 10%FBS<br>▪ DMEM/F12 + 10%FBS with ITS	*Fig. S7*	**DMEM + 10%FBS**

Fig. S2: Adrenoid formation: Ratio & Total cell number

A) Adrenoids were obtained by centrifugation (220g for 10 minutes), using a different number of total cells and a different ratio between cortical (H295R) and medullary (MTT) cells. **B)** A different number of H295R cells has been used to optimize the formation of spheroids composed of H295R alone.

Adrenoid and spheroid formation was monitored at 5 and 7 days, after 48 hour from induction. Scale bar: 200 µm

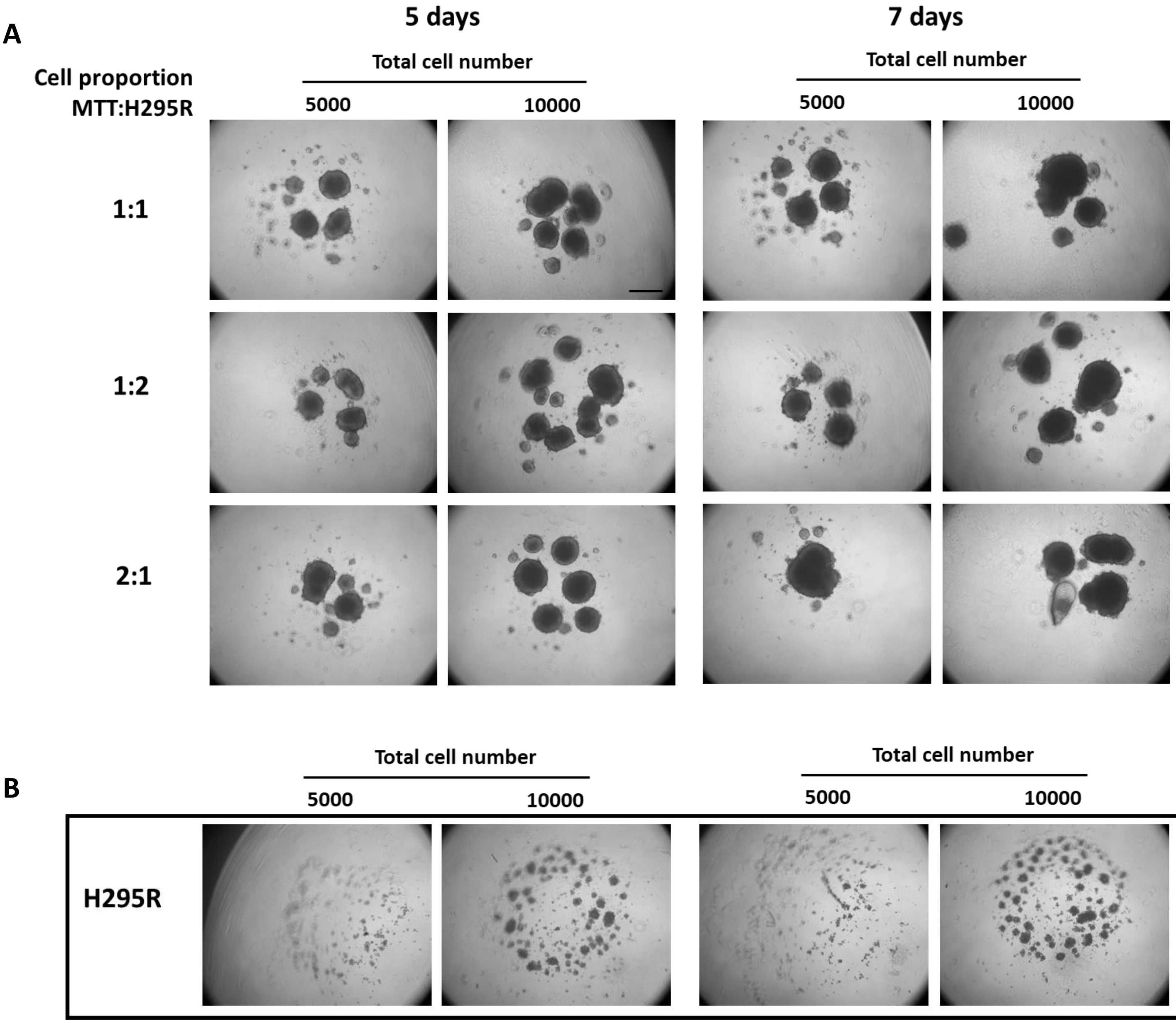

Fig. S3: Adrenoid formation: AGGREGATION METHODS 1 - SETTLING

Adrenoids were obtained by using 10^5 cells in a different ratio between the adrenocortical (H295R) and the medullary (MTT) cell line, leaving them to settle in the round-bottomed 96-well. Adrenoid/spheroid formation was monitored 48 hour after induction (upper panel) and after 96 hours (lower panel). Scale bar: 200 µm

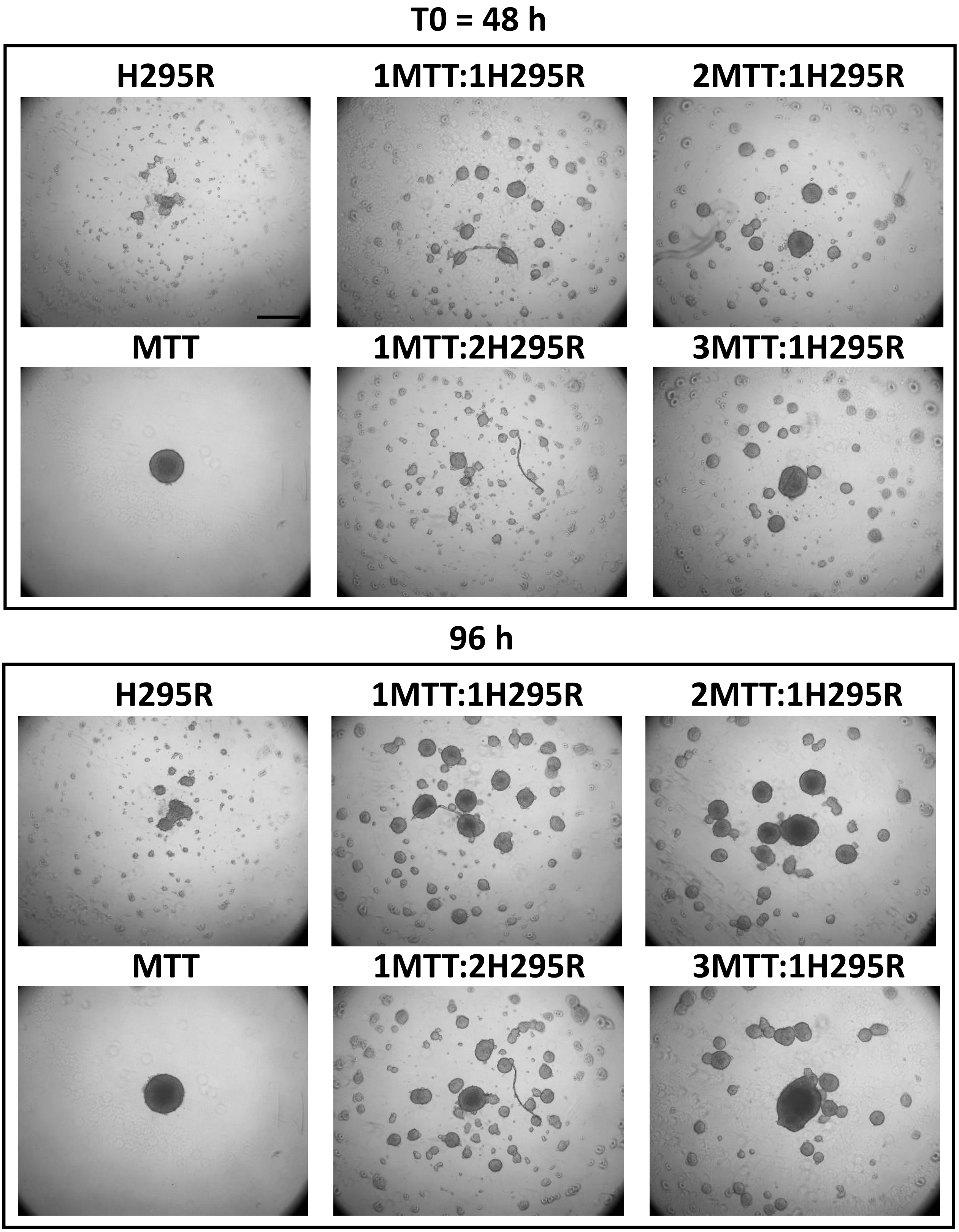

Fig. S4: Adrenoid formation: AGGREGATION METHODS 2 – ORBITAL SHAKER

Adrenoids were obtained by using 10^5 cells in a different ratio between the adrenocortical cell line (H295R) and the medullary (MTT), by agitation on orbital shaker for 2 hours in a 96well-round bottomed. Adrenoid/spheroid formation was monitored 48 hour after induction (upper panel) and after 96 hours (lower panel). Scale bar: 200 µm

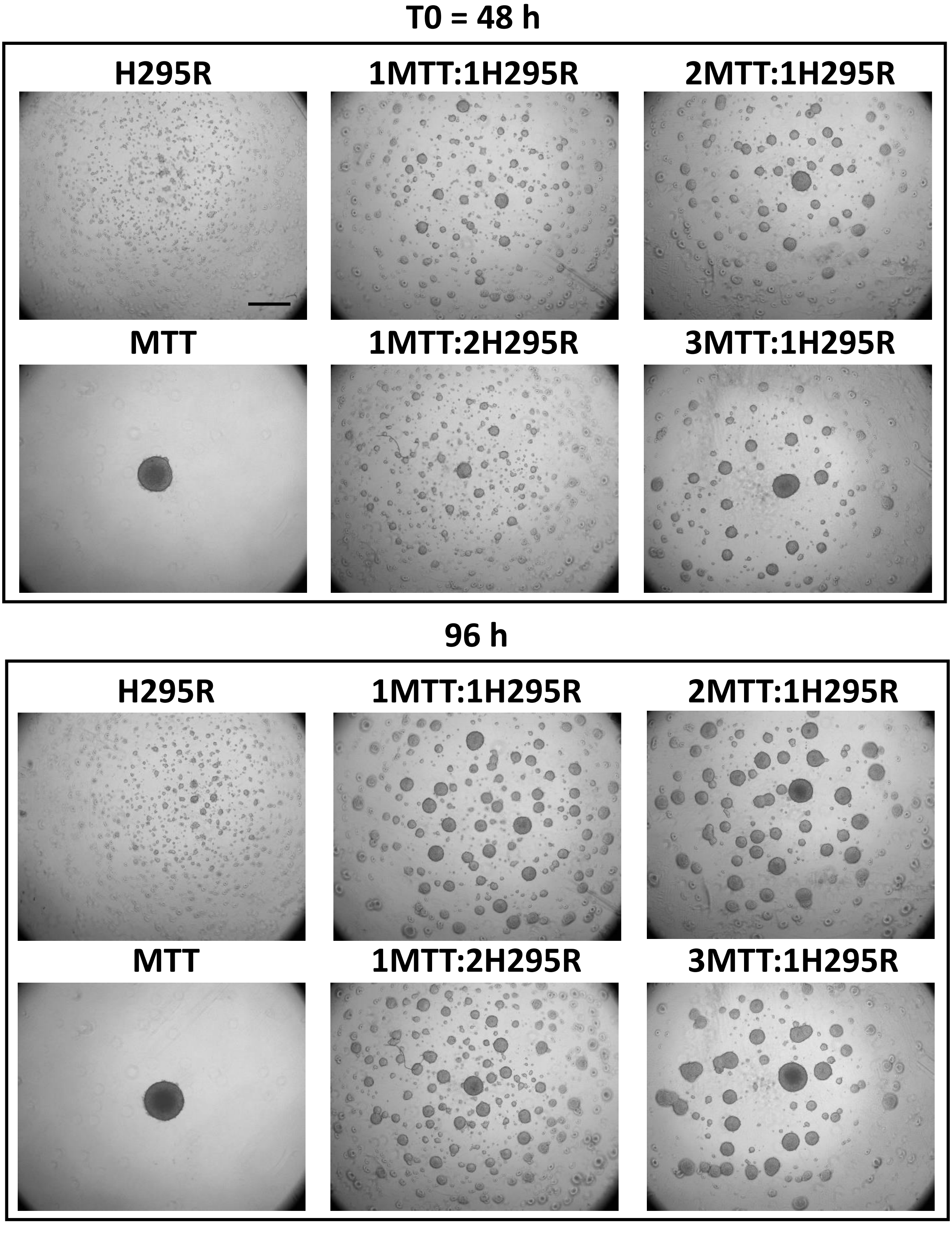

Fig. S5: Adrenoid formation: CENTRIFUGE TIMING
The speed and timing of the centrifuge (1000g for 5 minutes) was used to compare the formation of mixed Adrenoids with spheroids derived only from H295R. Adrenoid/spheroid development was monitored at 48 and 96 hours after induction. Scale bar: 200 µm

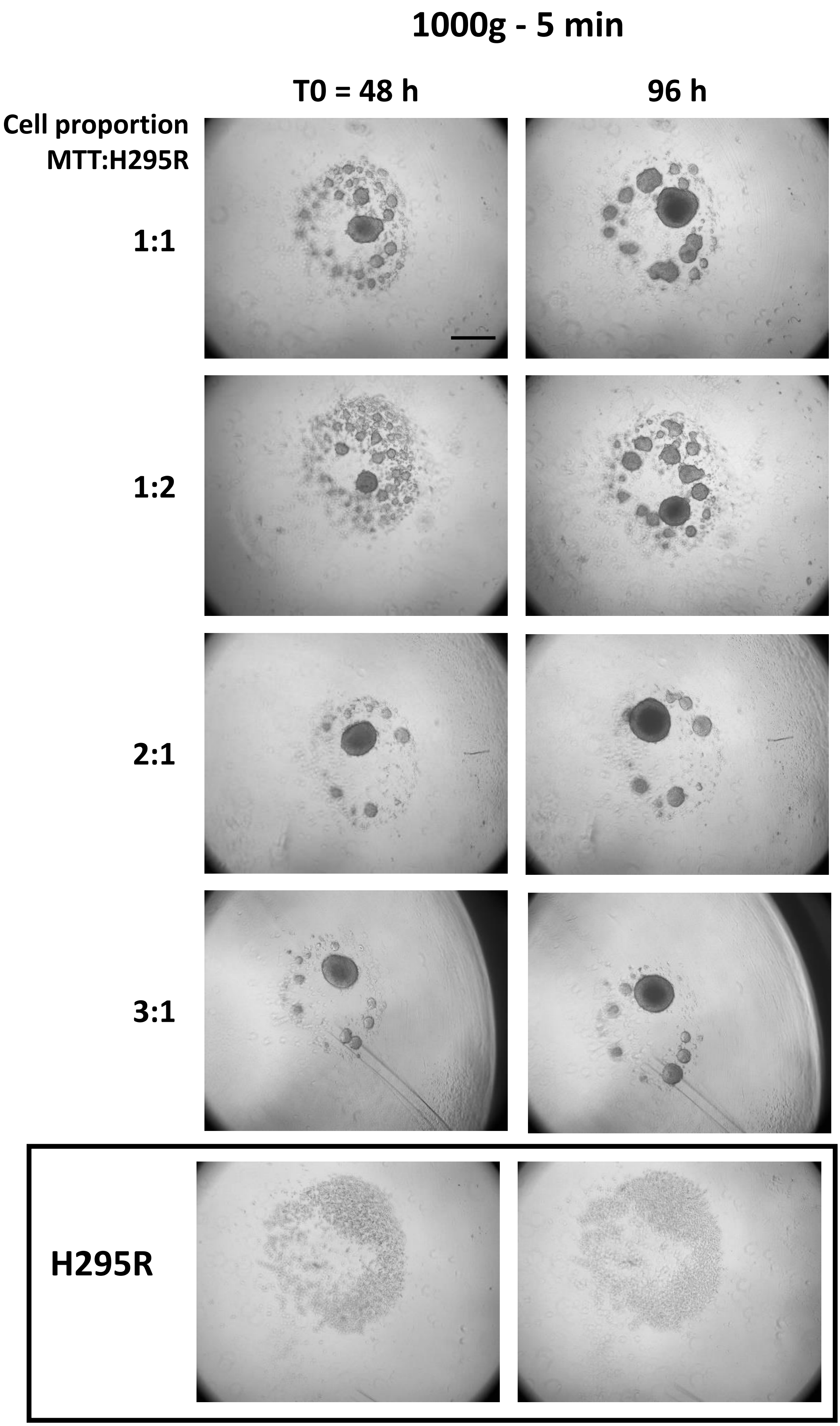

Fig. S6: Adrenoid formation: Timing of 3D formation
Adrenoids were obtained by centrifugation of $2x10^3$ total cells (1000g for 10 minutes), using different ratio between cortical (H295R) and medullary (MTT) cells. Adrenoid development/formation was monitored for 24-48-72 and 96 hours after induction. Scale bar: 400 µm

2000 cells - 1000g -10 min

Cell proportion MTT:H295R — **24 h**, **48 h**, **72 h**, **96 h**

1:1

2:1

3:1

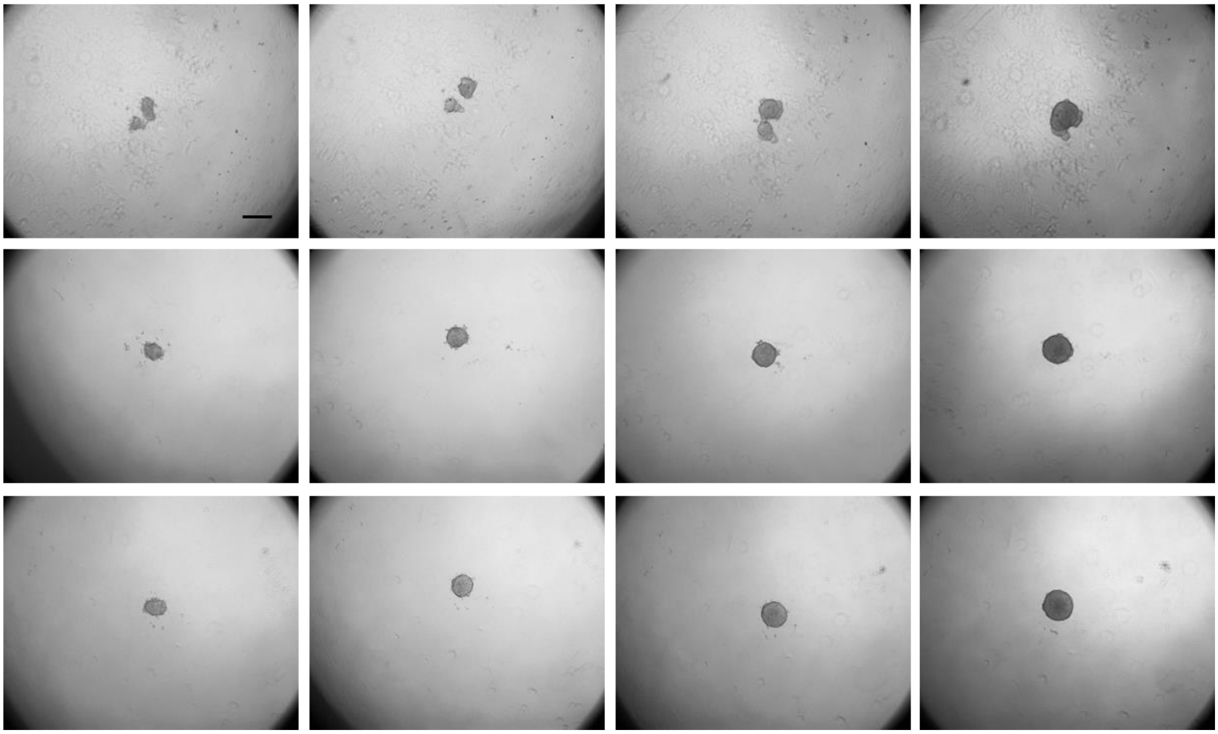

Fig. S7: Adrenoid formation: OPTIMIZATION OF CULTURE MEDIUM

For the formation of Adrenoids (final ratio 2MTT:1H295R), two different culture media were compared, DMEM + 10% FBS (specific for MTT) and DMEM/F12 + 10% FBS supplemented with ITS (specific for H295R). Adrenoid growth was followed after 48 hours from induction (T0) and after a further 72 hours. Scale bar: 200 µm

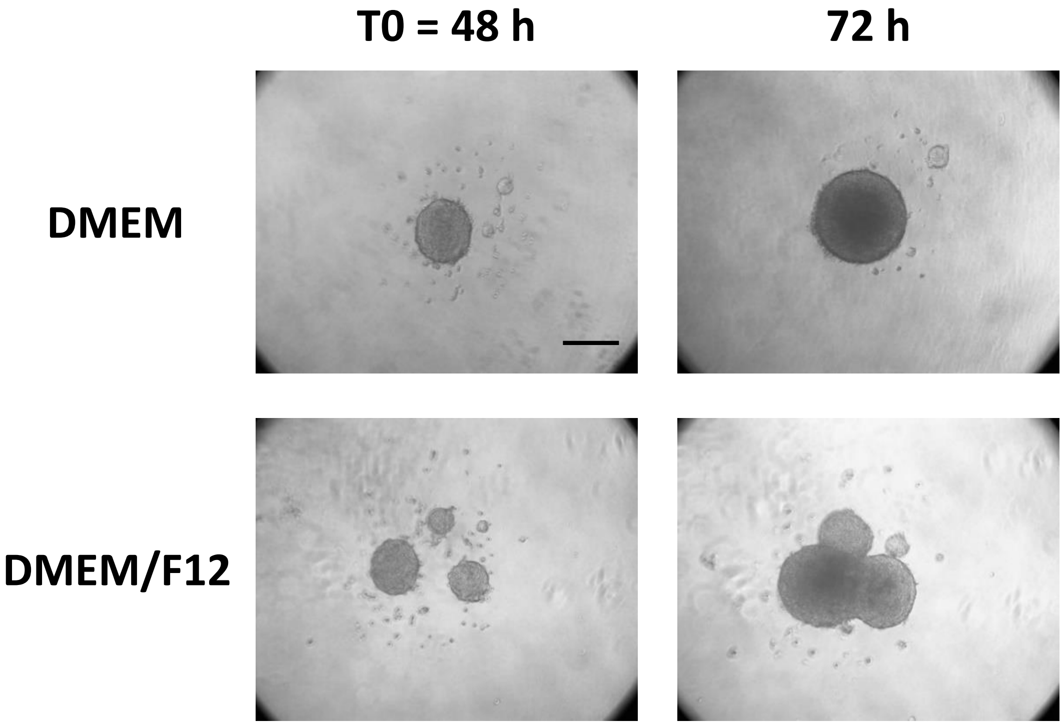

Fig. S8: Original images of blots and gels in Figures 7C and E

To allow multiple detection of proteins on the same membranes, they were cut prior to hybridization with the different antibodies during the blotting procedure. The corresponding fuller-length, original, unprocessed blots for each antibody are shown below as well as the UV acquisition of the Stain-free gel for total protein load/lane shown as loading control. Cut-out membranes were incubated with specific primary and secondary antibodies as detailed in the Materials and Methods' section (Anti-TH, anti-PNMT, anti-CYP11B1 and anti-STAR, as indicated below). Molecular markers are indicated in the cut blots and as total molecular ladder in the full length gels before membrane transfer and cutting.
Image acquisition, analysis and processing were performed with Image Lab software version 6.0 on a Chemi-Doc TM Touch instrument (Bio-Rad). Any processing was applied systematically to the acquired total image to maintain the relative intensity of bands in each lane.

Fig.7C upper panel (main manuscript)

Original unprocessed membrane and gel for total protein loading

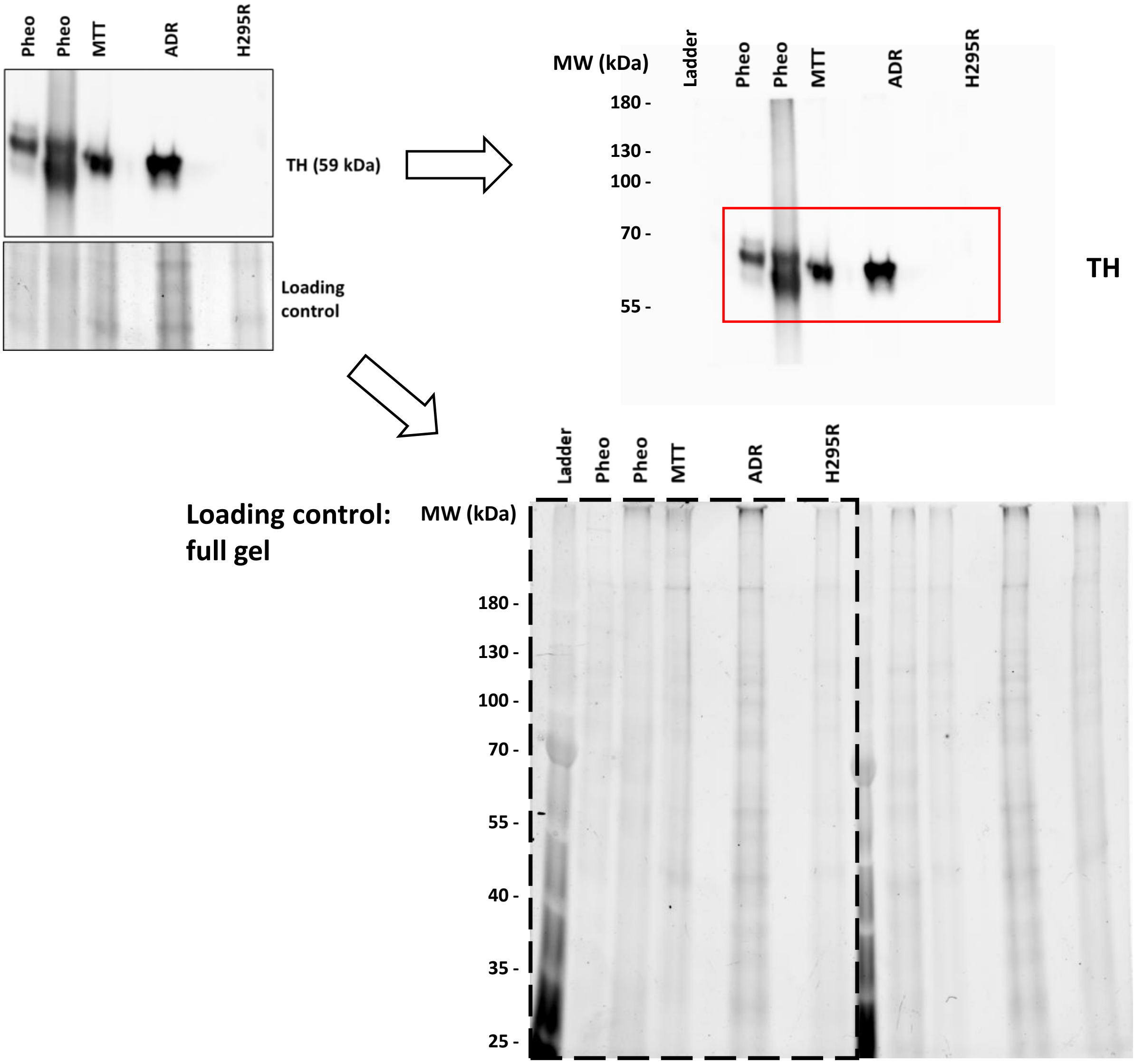

Original unprocessed membrane and gel for total protein loading

Fig.7C lower panel (main manuscript)

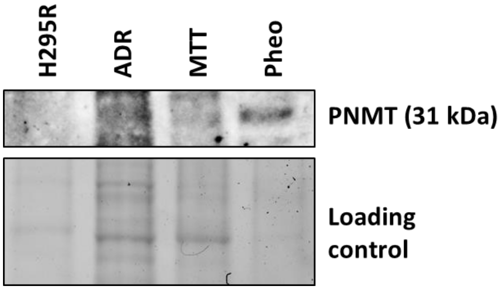

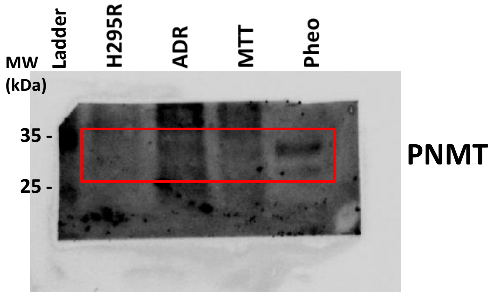

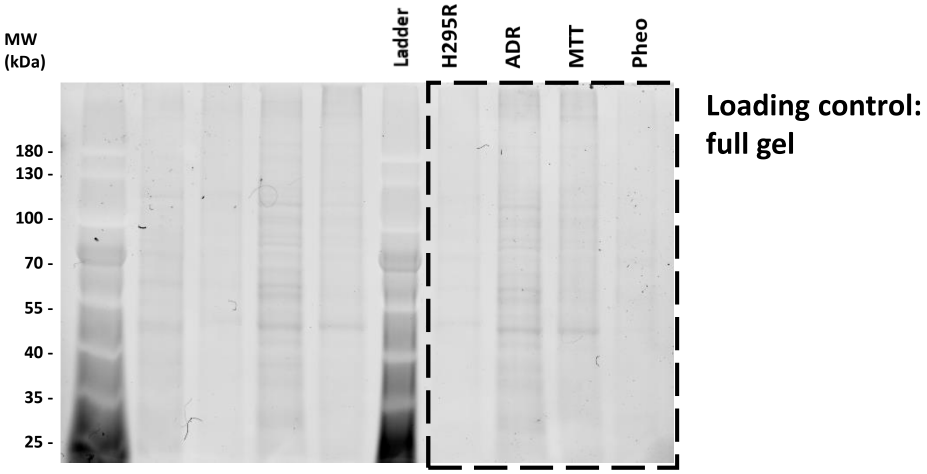

Original unprocessed membrane and gel for total protein loading

Fig.7E upper panel (main manuscript)

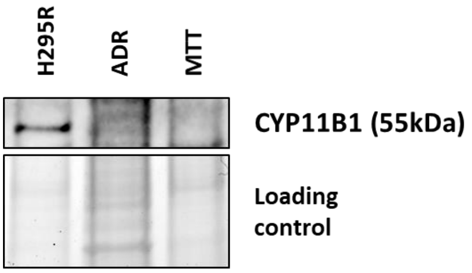

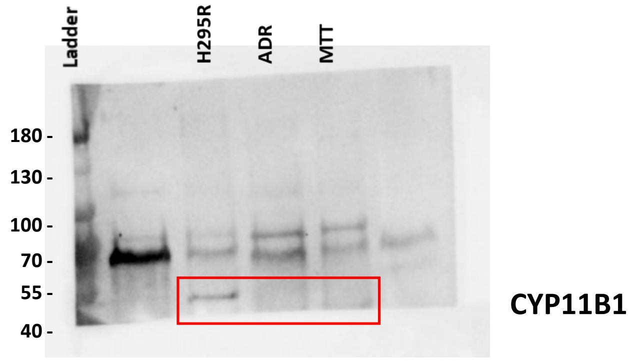

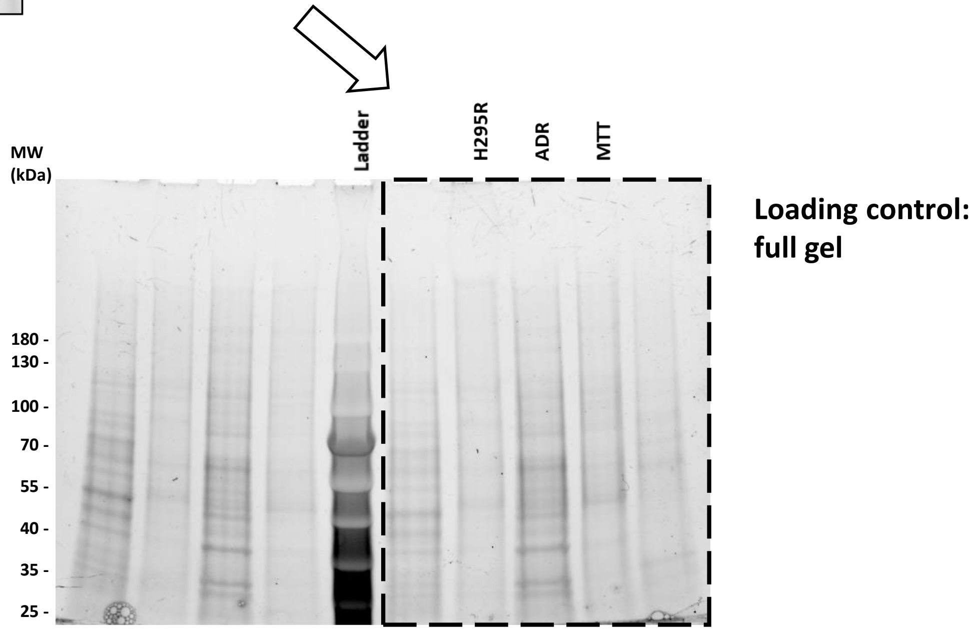

Fig.7E lower panel (main manuscript)

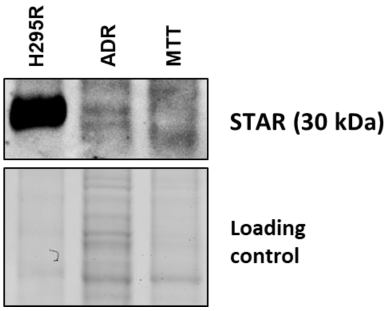

Original unprocessed membrane and gel for total protein loading

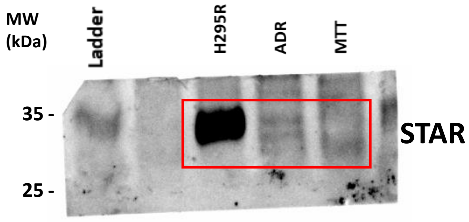

Loading control: full gel

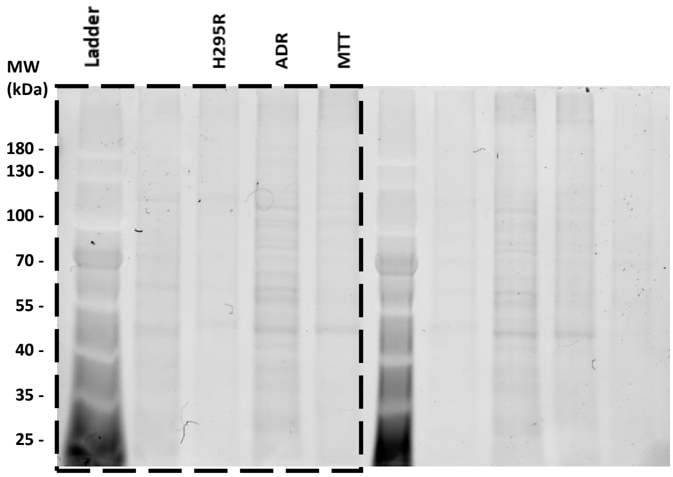

Supplement: Supplementary file 1 — Supplementary Information. [file 41598_2024_58664_MOESM1_ESM.pdf]
